# Serological survey reveals enzootic circulation of St. Louis encephalitis and West Nile viruses in semiarid Monte ecosystem of Argentina

**DOI:** 10.1038/s41598-024-55723-0

**Published:** 2024-02-29

**Authors:** Kevin A. Rucci, Diego L. Arias-Builes, Andrés M. Visintin, Adrián Diaz

**Affiliations:** 1 Laboratorio de Arbovirus, Instituto de Virología “Dr. J. M. Vanella”, Facultad de Ciencias Médicas, Universidad Nacional de Córdoba, Córdoba, Argentina; 2 Ministerio de Ciencia, Tecnología e Innovación, Consejo Nacional de Investigaciones Científicas y Técnicas (CONICET), Ciudad Autónoma de Buenos Aires, Argentina; 3 Departamento de Ciencias Básicas y Tecnológicas, Universidad Nacional de Chilecito, Chilecito, La Rioja Argentina; 4 Centro de Investigación e Innovación Tecnológica (CENIIT), Instituto de Biología de la Conservación y Paleobiología (IBICOPA), Universidad Nacional de La Rioja, La Rioja, Argentina; 5 Centro de Investigaciones Entomológicas de Córdoba, Instituto de Investigaciones Biológicas y Tecnológicas (IIByT), Facultad de Ciencias Exactas, Físicas y Naturales, Universidad Nacional de Córdoba, Córdoba, Argentina

**Keywords:** Ecological epidemiology, Viral infection

## Abstract

St. Louis encephalitis virus (SLEV) and West Nile virus (WNV) are arboviruses transmitted by *Culex* mosquitoes and amplified in avian hosts. The present study aimed to investigate the presence and seasonal circulation of SLEV and WNV in La Rioja province, within the semiarid ecoregion of the Monte, Argentina. Over a two-year period, avian sera were collected and tested for neutralizing antibodies against SLEV and WNV. Our results reveal the enzootic activity of both viruses in this challenging environment. SLEV seroprevalence was 4.5% (35/778), with higher activity in spring (2016) and autumn (2017). WNV seroprevalence was 3.5% (27/778), peaking during the summer 2016–2017. Greater seroprevalence for SLEV in 2016 was detected for the Lark-like Brushrunner (*Coryphistera alaudina*) and the Short-billed Canastero (*Asthenes baeri*) and in 2017 for the Black-crested Finch (*Lophospingus pusillus*) and Lark-like Brushrunner, whereas for WNV greater seroprevalence in 2016 was detected for the Picui Ground Dove (*Columbina picui*) and in 2017 for the Lark-like Brushrunner and Band-tailed Seedeater (*Catamenia analis*). Additionally, five avian individuals experienced seroconversion during the sampling period, namely the Lark-like Brushrunner and White-fronted Woodpecker (*Melanerpes cactorum*) for SLEV, and the Lark-like Brushrunner, Greater Wagtail Tyrant (*Stigmatura budytoides*) and Many-colored Chaco Finch (*Saltatricula multicolor*) for WNV. The study highlights the persistence and circulation of these viruses in a semiarid ecosystem, raising questions about overwintering mechanisms and transmission dynamics. This research contributes to understanding arbovirus ecology in diverse environments. Further investigations are needed to assess the specific mechanisms facilitating virus persistence in the Monte ecoregion.

## Introduction

St. Louis encephalitis (SLEV) and West Nile (WNV) viruses (*Flaviviridae*, *Flavivirus*) are two neurotropic arboviruses belonging to the Japanese encephalitis virus serocomplex which cause clinical manifestations, ranging from an asymptomatic or undifferentiated febrile condition to a febrile illness and encephalitis, rarely evolving to a more complex neuroinvasive condition^1^. Both viruses are maintained in nature by enzootic transmission networks between several species of *Culex* mosquitoes and different avian species, mainly Passeriformes and Columbiformes, as vectors and amplifying hosts, respectively^2,3^.

St. Louis encephalitis virus was first detected in St. Louis, Missouri, US in the summer of 1933, during an epidemic with more than 1000 cases within areas of the city adjacent to storm drains and sewage channels that generated high densities of *Culex* mosquitoes. Since its discovery, several outbreaks have occurred, the last major outbreak occurring in Florida during the summer of 1990^1^. Up to date, SLEV has been detected throughout the Americas, from Canada to Argentina. However, outbreaks in South America have been rare or sporadic^2^. Principal amplifying hosts in North America include house sparrows, house finches, blue jays, pigeons, and mourning doves^1^. In Argentina, SLEV reemerged in 2002 and produced the first encephalitis outbreak in Córdoba city in 2005, where 47 human cases were detected^4^. Later, a small new outbreak occurred in 2010 in the same city, as well as sporadic cases in several provinces in the northeast and center of Argentina^5,6^. In Argentina, SLEV has been isolated from humans (Buenos Aires province), *Culex* mosquitoes, and wild rodents (Córdoba), but never from birds. However, neutralizing antibodies have been detected in several families of wild birds (Furnariidae, Columbidae, Tyrannidae, Fringillidae, Icteridae, Ardeidae, and Cotingidae)^7^.

West Nile virus was first isolated in the West Nile district in Uganda in 1937, and subsequently also detected in other areas of Africa, Europe, South Asia, Australia, and America. In America, it was first detected in New York city in 1999, during an encephalomyelitis outbreak in humans. In South America, specific antibodies against WNV have been detected in birds and equines in the North (Colombia and Venezuela)^8–10^. Principal WNV hosts include house sparrows and robins^11^. In Argentina, WNV was first detected in 2006 in horses of Buenos Aires province, however, several studies indicate that it would have been introduced in late 2004, based on the detection of neutralizing antibodies in resident birds^12^.

The ability of both viruses to be transmitted and amplified by various species of *Culex* mosquitoes and avian hosts enables them to successfully colonize and establish themselves in diverse and novel biomes. For instance, following its introduction to the US, WNV effectively adapted and thrived across a broad spectrum of ecosystems, ranging from arid deserts (California, Arizona, New Mexico) and shrublands to pastures (Central Great Plains) and forests (northeast coast)^3^. A comparable expansive geographical distribution is observed for SLEV^1^. In Argentina, the majority of arbovirus studies have primarily focused on subtropical and temperate regions (such as Buenos Aires, Córdoba, Chaco, Tucumán, and Salta), which are anticipated to exhibit favorable conditions for arbovirus transmission and maintenance^7,12–15^. Recently, Batallán et al.^16^ have reported the circulation of both viruses by means of detection of neutralizing antibodies in wild birds in the Monte biogeographic region. This finding was unexpected since the Monte ecosystem is a semiarid region with limited water availability. We questioned if this detected circulation was because of an established enzootic transmission focus or represented sporadic circulation. Hence, we aimed to further expand the knowledge of SLEV and WNV circulation in this semiarid area in La Rioja province, employing a serological survey. To assess this, we focused on two aspects, studying the seasonal circulation of these viruses in wild bird communities, and investigating whether birds undergo seroconversion. This allowed us to determine if there exists an enzootic activity and establishment of these viruses in this challenging environment^16^.

## Materials and methods

### Study site

**Figure 1:**
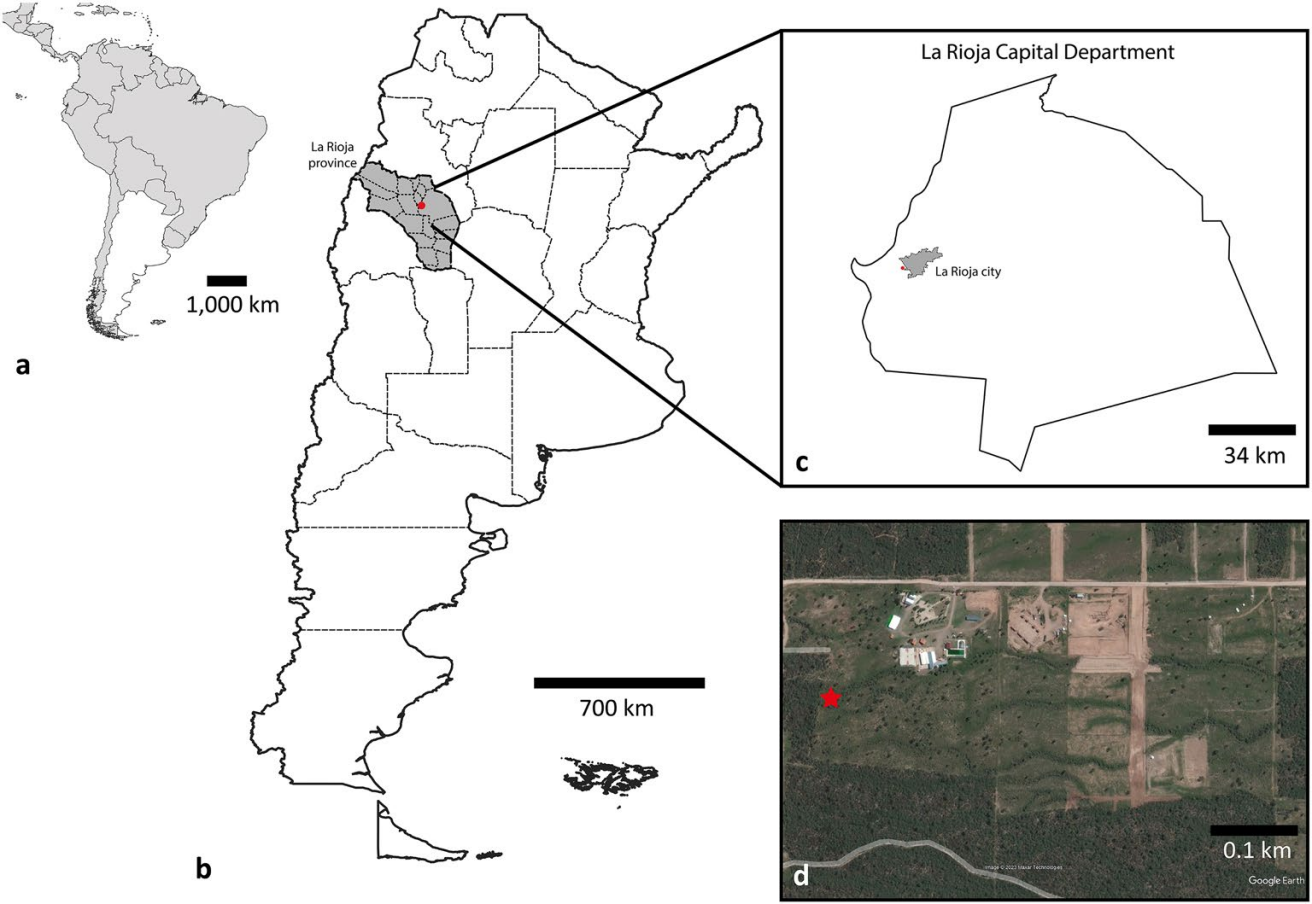
Sample site in the northwest region of Argentina. (**a**) Overview map of South America highlighting in grey the location of the Argentina Republic within the continent. (**b**) Argentine Republic, indicating in grey La Rioja province and as a red spot the sample site, “Camping del Club Legislativo La Rioja”. (**c**) La Rioja Capital department, with La Rioja city shaded in grey and the sample site as a red spot. (**d**) Landscape from the “Camping del Club Legislativo La Rioja”, extracted from Google Earth. Red star indicates sample site.

The study was conducted at “Camping del Club Legislativo La Rioja”, located in the vicinity of La Rioja city within La Rioja province, situated in the Argentine northwest region (29º 26 23 S; 66º 54 29 W; 620 masl.) (Fig. 1). Arana et al.^17^ described that a part of La Rioja belongs to the biogeographic region of Monte province, specifically to the Septentrional district. This area is characterized by an average annual precipitation of 200 to 400 mm, accumulated mainly during the warm season (October to March)^17,18^. Dominant vegetation is of the xerophytic type, which has developed a set of morphological, anatomical, and physiological adaptations to efficiently use the water and tolerate the temperatures of the region. Zonal vegetation (= climatic) is composed of a steppe of shrubs that do not exceed 3 m in height, characterized by scarcity of pasture and absence of trees. The most important shrub-steppe is the *jarillal*, formed by densely arranged shrubs of genus *Larrea* (*jarilla*), always characterized by abundant Zygophyllaceae and often featuring trees of the genus *Neltuma* (= *Prosopis*). The climate of this ecoregion does not allow the development of large forest masses and those present are edaphic communities forming riparian forests along rivers of permanent flow, which are dominated by *Neltuma nigra* (= *P. nigra*), *N. alpataco* (= *P. alpataco*), *N. argentina* (= *P. argentina*) (Fabaceae), and *Bulnesia retama* (Zygophyllaceae)^17,19^.

### Bird capture and sample collection

Bird collection was carried out using six mist nets (9 m length × 2.5 m height, 36 mm mesh) operated during sunrise (about 05:00 to 11:00 h depending on the season). Field sampling was performed during 4 days in each season (spring, summer, autumn, and winter) for 2 consecutive years (2016 and 2017). An alphanumeric aluminum band was placed on the tarsus to identify individuals in case of recapture, following the Centro Nacional de Anillado de Aves (National Center of Bird Banding) guidelines (https://www.csnat.unt.edu.ar/investigacion/institutos/cenaa). Bird species were identified by using a specialized field guide for bird species from Argentina and Uruguay^20^. The species and body mass of each bird were recorded. Prior to release, sampled birds were hydrated with sugar water. Blood was collected by jugular (for most species) or brachial (for columbids) venipuncture, using 27 G sterile needles. Birds weighing less than 10 g were not bled. Blood was collected in plastic tubes containing 0.45 mL or 0.9 mL (depending on the required sample volume: 100 µL or 200 µL, respectively) of phosphate buffered saline (PBS) for an approximate 1:10 serum dilution. The tubes were held at room temperature for 20–30 min to allow clotting, after which they were transferred to coolers. In the laboratory, samples were centrifuged at 5000*g* for 15 min for serum separation. Serum samples were stored at − 20 °C.

### Serological assays and data interpretation

Detection of neutralizing antibodies against SLEV and WNV was performed through Plaque-Reduction Neutralization Test (PRNT) in Vero Cl76 cell line (ATCC CRL-587). The screening test serum samples were incubated with 100 PFUs of each virus. Autochthonous low-passage SLEV CbaAr-4005 and WNV E/7229/06 strains were used. The SLEV CbaAr-4005 strain was isolated from *Culex quinquefasciatus* mosquitoes collected in Córdoba province^21^, whereas the WNV E/7229/06 strain was isolated from a dead horse in Buenos Aires province, Argentina^22^. Positive control corresponded to a known serum with neutralizing antibodies for each virus, while negative control was represented by a serum without neutralizing antibodies. Samples were analyzed with an initial dilution of 1:20. All serum samples that neutralized 80% or more of the inoculated plaque-forming units at dilutions ≥ 1:20 were considered positive. Samples positive for both viruses were subjected to titration. Seven serial two-fold dilutions of serum were prepared, resulting in final dilutions of 1:20, 1:40, 1:80, 1:160 and 1:320. Endpoint titers were assigned as the reciprocal of the greatest dilution in which > 80% neutralization of the challenge virus was observed. Seroconversion was defined as a bird that was seronegative when first captured and became seropositive at recapture. Seroreversion was defined as a seropositive bird whose antibodies decreased below the cut-off value of 20 at recapture^23^. Serological data were interpreted following the same criteria explained by Diaz et al.^7^.

### Statistical analysis

Seroprevalence with its 95% confidence interval was calculated for both SLEV and WNV using the *binom* library and the Pearson-Kloper method in R^24^. Proportions were considered for each season, year, and species (with sample size > 10 individuals). The effects of season, year, and avian species were assessed using Generalized Linear Models with binomial error distribution and logit link function. We used the presence and absence of neutralizing antibodies for both viruses, SLEV and WNV, as binary response variables. Every single explanatory variable was modeled as a main effect and in combination as additive effects. To select those models that explained the greatest variability of the response variable, we employed the second-order Akaike information criterion (AICc) for small samples. The support for each model was evaluated using the ΔAICc, defined as the difference in AICc values between the model with the minimum AICc and the AICc of the model under consideration. We selected the best model as the one with a ΔAICc < 2. Additionally, Akaike weights (ω_i_) and evidence ratio (ER), which is the ratio between the weight of the best model and the second best, were calculated as additional levels of support for the best models^25^. Model selection was carried out using the *AICcmodavg* library in R^26^. All analyses were conducted in R Studio statistical software, v.4.2.1^27^.

### Ethical approval

The capture, manipulation, blood collection, and transport of samples were approved by Fauna Silvestre and the Secretaría de Ambiente of La Rioja province (resolution S.A. 0215/13). The study was performed in accordance with relevant guidelines and regulations for the use of birds in research developed by the Ornithological Council (https://www.aaalac.org/pub/?id=E9019213-EE55-98AB-F68E-EF2B10C31360) and in accordance with the U.K. Animals (Scientific Procedures) Act, 1986 and associated guidelines, EU Directive 2010/63/EU for animal experiments. All methods and procedures complied with the ARRIVE guidelines for reporting animal research.

## Results

### Seasonal circulation of SLEV and WNV

A total of 778 sera belonging to 51 avian species from 17 families were subjected to PRNT for detection of neutralizing antibodies against SLEV and WNV. The most frequently captured birds in each season were the tanagers (family Thraupidae), which represented 30.4% and 50% in winter and spring 2016, respectively, and 54.9% and 67.1% in autumn and winter 2017, respectively. The Columbidae (15.7%) and Furnariidae (13.2%) families were most prevalent during the summer of 2016–2017. Conversely, the family Emberizidae (buntings) showed high representation in winter 2016 (28.3%) and autumn 2017 (23.7%).

**Figure 2:**
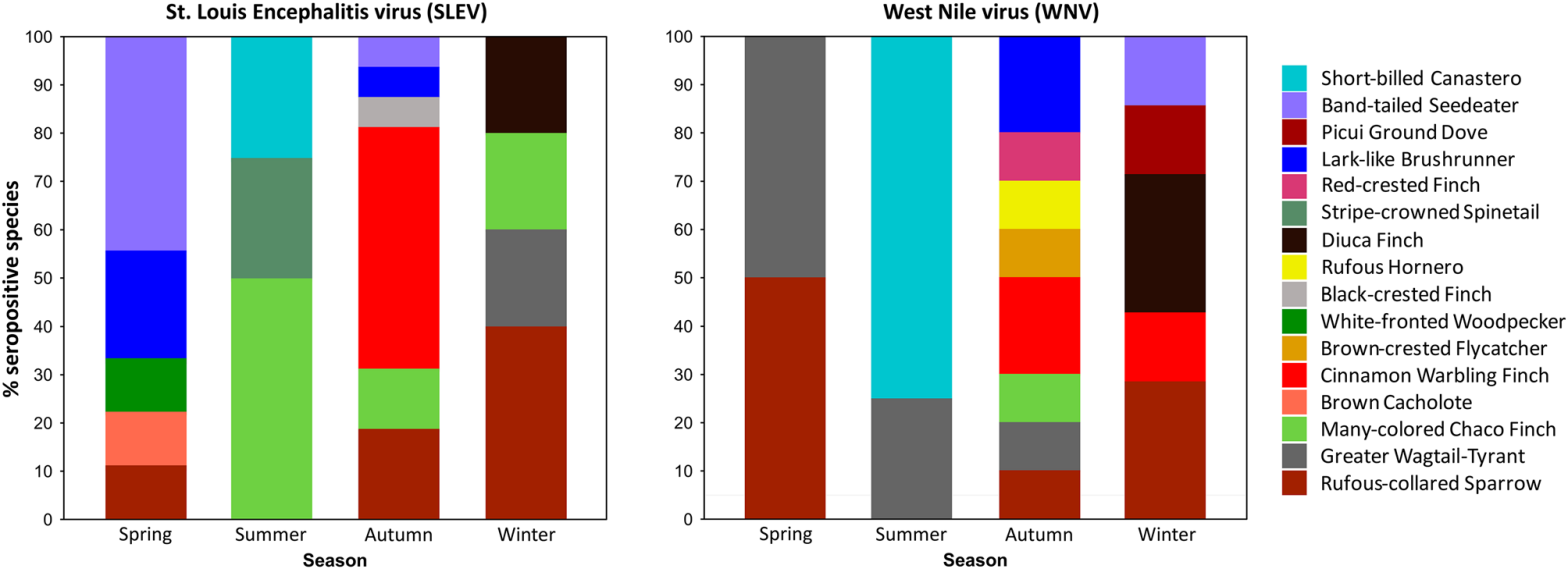
Seasonal species composition seroprevalence of St. Louis encephalitis virus (SLEV) (left) and West Nile virus (WNV) (right) for the period 2016–2017 in La Rioja, Argentina.

The best model, selected by the ΔAICc criterion, for SLEV seroprevalence, included the variables season and year (ω_i_ = 0.85, ER = 13.51) (Table 1). Overall, SLEV seroprevalence accounted for 4.5% (35/778), being higher in 2016 (5.68%, 17/299) than in 2017 (3.76%, 18/479). Regarding seasonal variation, two peaks of seroprevalence were observed: one in spring 2016 (5.26%, 9/171) and another in autumn 2017 (6.22%, 16/257). Positivity for winter 2016 (4.35%, 4/92) and summer 2016–2017 (4.13%, 5/121) were similar, with a marked decline towards winter 2017 (0.73%, 1/137) (Table 2). The most seropositive bird species during 2016 were the Lark-like Brushrunner (*Coryphistera alaudina*) (14.29%, 2/14) and the Short-billed Canastero (*Asthenes baeri*) (7.14%, 1/14), both resident (i.e., non-migratory) birds. During the following year, the most seroprevalent birds were the resident Black-crested Finch (*Lophospingus pusillus*) (8.33%, 1/12) and the Lark-like Brushrunner (7.69%, 1/13) (Table 3, Fig. 2).

**Table 1:** Results of model selection of logistic regression models for St. Louis encephalitis (SLE) and West Nile (WN) viruses seroprevalence during 2016–2017 in La Rioja, Argentina.

Model	K	AICc	ΔAICc	ω_i_
SLEV				
Season + year	6	272	0.00	0.85
Season	5	277	5.21	0.06
Null	1	278	5.78	0.05
Year	2	278	6.42	0.03
Season + species	56	354	82.01	0.00
Species	51	358	86.43	0.00
Species + year	52	359	87.39	0.00
WNV				
Year	2	203	0.00	0.81
Null	1	207	3.77	0.12
Season	5	209	5.66	0.05
Season + year	6	210	7.18	0.02
Species + year	52	273	70.43	0.00
Species	51	279	75.82	0.00
Season + species	56	282	78.98	0.00

The selected model was the one with the least ΔAICc.

*K* degrees of freedom, *ω*_i_ Akaike weight.

**Table 2:** SLEV and WNV neutralizing antibody prevalence among all bird species combined, by season, La Rioja, Argentina.

Season	SLEV		WNV	
	Pos/test	%[IC95]	Pos/test	%[IC95]
Winter (2016)	4/92	4.35 [1.20–10.76]	1/92	1.09 [0.03–5.90]
Spring (2016)	9/171	5.26 [2.43–9.75]	2/171	1.17 [0.14–4.16]
Summer (2016–2017)	5/121	4.13 [1.35–9.38]	8/121	6.61 [2.90–12.61]
Autumn (2017)	16/257	6.22 [3.60–9.91]	10/257	3.90 [1.88–7.03]
Winter (2017)	1/137	0.73 [0.018–4.00]	6/137	4.38 [1.62–9.29]
Total	35/778	4.50 [3.15–6.20]	27/778	3.47 [2.30–5.00]

**Table 3:** SLEV and WNV seroprevalence among bird species, by year, La Rioja, Argentina.

Family	Species	Migratory status	2016				2017			
			SLEV		WNV		SLEV		WNV	
			Pos/test	%[IC95]	Pos/test	%[IC95]	Pos/test	%[IC95]	Pos/test	%[IC95]
Icteridae	Grayish Baywing (*Agelaioides badius*)	Resident	0/13	0 [0–24.71]	0/13	0 [0–24.71]	–	–	–	–
Furnariidae	Short-billed Canastero (*Asthenes baeri*)	Resident	1/14	7.14 [0.18–33.87]	0/14	0 [0–23.16]	–	–	–	–
Thraupidae	Band-tailed Seedeater (*Catamenia analis*)	Resident	4/63	6.35 [1.76–15.47]	0/63	0 [0–5.69]	1/14	7.14 [0.18–33.87]	1/14	7.14 [0.18–33.87]
Columbidae	Picui Ground Dove (*Columbina picui*)	Resident	0/14	0 [0–23.16]	1/14	7.14 [0.18–33.87]	0/34	0 [0–10.28]	0/34	0 [0–10.28]
Furnariidae	Lark-like Brushrunner (*Coryphistera alaudina*)	Resident	2/14	14.29 [1.78–42.81]	0/14	0 [0–23.16]	1/13	7.69 [0.19–36.03]	2/13	15.38 [1.92–45.45]
Thraupidae	Diuca Finch (*Diuca diuca*)	Resident	–	–	–	–	1/38	2.63 [0.07–13.81]	2/38	5.26 [0.64–17.75]
Thraupidae	Black-crested Finch (*Lophospingus pusillus*)	Resident	–	–	–	–	1/12	8.33 [0.21–38.48]	0/12	0 [0–26.46]
Thraupidae	Cinnamon Warbling Finch (*Poospiza ornata*)	Resident	–	–	–	–	8/119	6.72 [2.95–12.82]	3/119	2.52 [0.52–7.19]
Thraupidae	Many-colored Chaco Finch (*Saltatricula multicolor*)	Resident	3/45	6.67 [1.40–18.27]	0/45	0 [0–7.87]	2/45	4.44 [0.54–15.15]	1/45	2.22 [0.06–11.77]
Passerellidae	Rufous-collared Sparrow (*Zonotrichia capensis*)	Resident	3/44	6.82 [1.43–18.66]	1/44	2.27 [0.06–12.02]	3/87	3.45 [0.72–9.75]	3/87	3.45 [0.72–9.75]
TOTAL	–	–	13/207	6.28 [3.39–10.5]	2/207	0.97 [0.12–3.45]	17/362	4.7 [2.76–7.41]	12/362	3.31 [1.72–5.72]

Only species with sample size > 10 are shown.

Seropositive species with sample size < 10. 2016 = *Cranioleuca pyrrhophia* (1/2), *Melanerpes cactarum* (1/7), *Pseudoseisura lophotes* (1/5), *Stigmatura budytoides* (1/9). 2017 = no positive species.

Seronegative species with sample size < 10. 2016 = *Agriornis Micropterus* (0/1), *Ammodramus humeralis* (0/2), *Anairetes flavirostris* (0/1), *Cyclarhis gujanensis* (0/1), *Diuca diuca* (0/4), *Elaenia parvirostris* (0/4), *Falco sparverius* (0/4), *Furnarius rufus* (0/1), *Knipolegus aterrimus* (0/1), *Leptasthenura platensis* (0/1), *Leptotila verreauxi* (0/1), *Lophospingus pusillus* (0/2), *Molothrus bonariensis* (0/2), *Molothrus rufoaxillaris* (0/4), *Myiarchus tyrannulus* (0/3), *Passer domesticus* (0/1), *Pitangus sulphuratus* (0/1), *Poospiza melanoleuca* (0/6), *Poospiza ornata* (0/2), *Poospiza torquata* (0/1), *Saltator aurantiirostris* (0/2), *Synallaxis albescens* (0/7), *Troglodytes aedon* (0/8), *Turdus amaurochalinus* (0/3), *Turdus chiguanco* (0/1), *Tyrannus melancholicus* (0/1), *Veniliornis mixtus* (0/2), *Vireo olivaceus* (0/1), *Zenaida auriculata* (0/2). 2017 = *Agelaioides badius* (0/9), *Anairetes flavirostris* (0/1), *Asthenes baeri* (0/9), *Athene cunicularia* (0/5), *Coccyzus melacoryphus* (0/1), *Colaptes melanochloros* (0/1), *Coryphospingus cucullatus* (0/2), *Elaenia parvirostris* (0/3), *Embernagra platensis* (0/2), *Furnarius rufus* (0/2), *Melanerpes cactarum* (0/2), *Mimus patagonicus* (0/2), *Myiarchus tyrannulus* (0/2), *Myioborus brunniceps* (0/1), *Nothura maculosa* (0/2), *Patagioenas maculosa* (0/5), *Poospiza melanoleuca* (0/2), *Pseudoseisura lophotes* (0/1), *Saltator aurantiirostris* (0/4), *Spiziapteryx circumcincta* (0/1), *Stigmatura budytoides* (0/4), *Troglodytes aedon* (0/4), *Turdus amaurochalinus* (0/2), *Turdus chiguanco* (0/4), *Veniliornis mixtus* (0/2), *Vireo olivaceus* (0/1), *Xolmis irupero* (0/1), *Zenaida auriculata* (0/3).

For WNV, the best model included only the year variable (ω_i_ = 0.81, ER = 6.58). Overall, 3.5% (27/778) of the total were positive for this virus, with higher seroprevalence in 2017 (4.8%, 23/479) than in 2016 (1.34%, 4/299). Although season was not selected as an explanatory variable, it could be observed a seasonal pattern in WNV seroprevalence. The lowest seroprevalences were recorded in winter (1.09%, 1/92) and spring (1.17%, 2/171) 2016, rising to a peak in summer 2016–17 (6.61%, 8/121) and decreasing but maintaining high levels towards autumn (3.9%, 10/257) and winter (4.38%, 6/137) 2017 (Table 2). The most frequently positive bird species in 2016 was the Picui Ground Dove (*Columbina picui*) (7.14%, 1/14), and in 2017 was the Lark-like Brushrunner (15.38%, 2/13) and the Band-tailed Seedeater (*Catamenia analis*) (7.14%, 1/14), all resident avian species (Table 3, Fig. 2).

### Seroconversion survey

From all captured birds, 36 were recaptured, being 34 of these seronegative for both SLEV and WNV at the first sampling event. Among these, 14% (5/34) underwent seroconversion during the sampling period. For SLEV, birds that seroconverted were the Lark-like Brushrunner (from June to October 2016) and the White-fronted Woodpecker (*Melanerpes cactorum*) (from September to November 2016). In the case of WNV, the birds that seroconverted included the Lark-like Brushrunner (from November 2016 to January 2017), the Greater Wagtail-Tyrant (*Stigmatura budytoides*) (from September to December 2016), and the Many-colored Chaco Finch (*Saltatricula multicolor*) (from April to May 2017). Additionally, two birds seroreversed, namely the Many-colored Chaco Finch (from December 2016 to December 2017) for SLEV and the Rufous Hornero (*Furnarius rufus*) (from April to June 2017) for WNV. Lastly, two birds were positive during their first capture and recapture event, the many-colored Chaco Finch (from December 2016 to March 2017) for SLEV and the Short-billed Canastero (from January to February 2017) for WNV.

## Discussion

St. Louis encephalitis and West Nile viruses are multi-host/multi-vector flaviviruses, endowing them with the capacity to be amplified and transmitted by a variety of avian and *Culex* mosquito species^28^. This ecological adaptability has facilitated their wide geographic distribution and colonization of numerous ecoregions. In Argentina, both viruses have been identified across diverse ecological zones, encompassing subtropical forests, thorned forests, shrublands, pastures, agricultural areas, and urbanized regions^7,12,15,29,30^. Intriguingly, Batallán et al.^16^ detected the presence of both viruses in semiarid ecosystems within the Monte region (La Rioja province), as evidenced by the identification of neutralizing antibodies in wild birds. Their study reported a lower seroprevalence for both viruses compared to our findings. Specifically, Batallán et al.^16^ recorded an overall seroprevalence of 2.4% for SLEV and 0.8% for WNV, in contrast to our results of 4.5% and 3.5%, respectively. Notably, the Cinnamon Warbling Finch (*Poospiza ornata*) exhibited higher seropositivity to SLEV, whereas the White-tipped Dove (*Leptotila verreauxi*) and Grayish Baywing (*Agelaioides badius*) displayed greater seroprevalence to WNV. Nevertheless, the seroprevalence of these viruses was lower compared to other regions in Argentina^7,12,30^. This reduced avian host seropositivity rate may stem from the relatively low mosquito population abundance in the semiarid Monte ecosystem. The mosquito community within this ecosystem is characterized by various *Culex* mosquito species, among which the most abundant are *Cx. apicinus*, *Cx. bidens, Cx. coronator*, *Cx. dolosus, Cx. interfor, Cx. maxi, Cx. pipiens, Cx. quinquefasciatus, Cx. saltanensis* and *Cx. tramazayguesi*^31–33^. These species, in turn, can act as vectors for SLEV and WNV^34,35^. Mosquito populations in the Monte ecoregion show remarkable stationary population dynamics with population peaks primarily occurring at the beginning of the warm season (October-December), and at the end of the same season (March–April), corresponding to periods of increased rainfall.

One of the most noteworthy attributes of vector-borne diseases is their distinct seasonality, intricately linked with the biology of their vectors. Consequently, the pathogens they carry must employ mechanisms to persist through adverse periods, adapting to the seasonal dynamics of their vectors (overwintering)^36^. Conceptually, three distinct overwintering mechanisms could come into play: (i) sustained yet diminished transmission, linked to the survival and residual biting activity of adult vectors, (ii) persistence within vector/hosts, and (iii) survival within the resistant stages of the vector^36^. Sustained transmission might occur in regions where vectors continue to bite hosts throughout the year. In these areas, the elongated interval between blood meals due to winter temperatures, coupled with an extended extrinsic incubation period (latency period), leads to infrequent clinical cases that may easily go unnoticed (as observed in the persistence of the *Culex*/West Nile virus system in southern California)^37^. It has been observed that Argentine populations of *Cx. quinquefasciatus* do not enter diapause during unfavorable periods (winter), remaining physiologically active, which allows for low but steady vectorial transmission^38^. The persistence of the virus within an avian host could result from a prolonged presence in tissues, subsequently leading to resurgence (as observed in West Nile virus infections in house sparrows)^39^. In the *Culex*/Western equine encephalitis virus system, a reemergence of viremia was experimentally observed in snakes following hibernation^40^. Furthermore, a pathogen can persist by establishing itself within the overwintering stages of the vector through vertical transmission, either in eggs or in newly emerged adults. This phenomenon is exemplified by the *Culex*/St. Louis encephalitis virus system and the *Aedes triseriatus*/La Crosse virus system, where persistence is maintained respectively in the eggs and the recently emerged larval/adult mosquitoes^41,42^. Persistence in unfavorable environments could also be explained by the repeated annual introduction of the virus through the dispersal by migratory birds^12^. SLEV and WNV are flaviviruses amplified by birds that could be dispersed by this type of host. It would be interesting to study the phylodynamics of these viruses in the Monte ecosystem to obtain evidence about the potential annual introduction or local establishment of viral strains. The consistent presence of antibodies against SLE and WN viruses in different resident bird species over a two-year sampling period, along with the observed seroconversions in resident birds (Lark-like Brushrunner, White-fronted Woodpecker, Greater Wagtail Tyrant, Many-colored Chaco Finch) at different times of the year, confirms the enzootic activity and establishment of these viruses within this challenging environment.

Our findings unequivocally affirm the enzootic establishment and activity of SLE and WN viruses within an ecosystem traditionally deemed unfavorable for arbovirus transmission, as exemplified by the Monte ecoregion. Consequently, future research endeavors aimed at pinpointing the specific overwintering mechanisms driving in this ecosystem are imperative.

## Data Availability

The datasets generated during and/or analyzed during the current study are available from the corresponding author upon reasonable request.
